# Loss of S1P Lyase Expression in Human Podocytes Causes a Reduction in Nephrin Expression That Involves PKCδ Activation

**DOI:** 10.3390/ijms24043267

**Published:** 2023-02-07

**Authors:** Faik Imeri, Bisera Stepanovska Tanturovska, Roxana Manaila, Hermann Pavenstädt, Josef Pfeilschifter, Andrea Huwiler

**Affiliations:** 1 Institute of Pharmacology, Inselspital, INO-F, University of Bern, CH-3010 Bern, Switzerland; 2 Medizinische Klinik D, University Hospital Münster, D-48149 Münster, Germany; 3 Pharmazentrum Frankfurt/ZAFES, University Hospital, Goethe University Frankfurt am Main, Theodor-Stern Kai 7, D-60590 Frankfurt am Main, Germany

**Keywords:** sphingosine 1-phosphate, sphingosine 1-phosphate lyase, glomerular disease, nephrotic syndrome, podocytes, Wilms tumor suppressor gene 1, nephrin, protein kinase Cdelta

## Abstract

Sphingosine 1-phosphate (S1P) lyase (SPL, *Sgpl1*) is an ER-associated enzyme that irreversibly degrades the bioactive lipid, S1P, and thereby regulates multiple cellular functions attributed to S1P. Biallelic mutations in the human *Sglp1* gene lead to a severe form of a particular steroid-resistant nephrotic syndrome, suggesting that the SPL is critically involved in maintaining the glomerular ultrafiltration barrier, which is mainly built by glomerular podocytes. In this study, we have investigated the molecular effects of SPL knockdown (kd) in human podocytes to better understand the mechanism underlying nephrotic syndrome in patients. A stable SPL-kd cell line of human podocytes was generated by the lentiviral shRNA transduction method and was characterized for reduced SPL mRNA and protein levels and increased S1P levels. This cell line was further studied for changes in those podocyte-specific proteins that are known to regulate the ultrafiltration barrier. We show here that SPL-kd leads to the downregulation of the nephrin protein and mRNA expression, as well as the Wilms tumor suppressor gene 1 (WT1), which is a key transcription factor regulating nephrin expression. Mechanistically, SPL-kd resulted in increased total cellular protein kinase C (PKC) activity, while the stable downregulation of PKCδ revealed increased nephrin expression. Furthermore, the pro-inflammatory cytokine, interleukin 6 (IL-6), also reduced WT1 and nephrin expression. In addition, IL-6 caused increased PKCδ Thr^505^ phosphorylation, suggesting enzyme activation. Altogether, these data demonstrate that nephrin is a critical factor downregulated by the loss of SPL, which may directly cause podocyte foot process effacement as observed in mice and humans, leading to albuminuria, a hallmark of nephrotic syndrome. Furthermore, our in vitro data suggest that PKCδ could represent a new possible pharmacological target for the treatment of a nephrotic syndrome induced by SPL mutations.

## 1. Introduction

Podocytes are visceral epithelial cells of the renal glomerulus that play a key role in glomerular blood filtration. These cells are highly differentiated and typically have a voluminous cell body, from where primary extensions radiate toward the capillaries with which they are associated and build a tight network of interdigitating foot processes. These foot processes interact with each other and with the foot processes of neighboring cells, thereby forming the slit diaphragm, which represents the basic ultrafiltration barrier of the kidney. The slit diaphragm is built by a complex of proteins that interact with each other in a homophilic and heterophilic manner and also interact with the actin cytoskeleton, thereby regulating cell morphology and actin dynamics [1,2]. Members of this complex include nephrin (NPHS1), podocin (NPHS2), NEPH1, CD2AP, zonula occludens 1, and P-cadherin [1,2]. Interrupting the normal interaction between these proteins, in one way or another, leads to a process called foot process effacement, which is characterized by the detachment of foot processes from the glomerular basement membrane and, ultimately, to the leakage of larger proteins, such as albumin, into the urine.

More than 50 gene mutations encoding for glomerular podocyte proteins have been described in various nephrotic syndrome pathologies [3,4]. Among these, several mutations were found in the *Sgpl1* gene, which encodes for sphingosine 1-phosphate (S1P) lyase (SPL), an enzyme that degrades S1P [5,6,7]. These patients develop a SPL insufficiency syndrome (SPLIS), which is manifested by steroid-resistant nephrotic syndrome, ichthyosis/acanthosis, primary adrenal insufficiency, immunodeficiency, and neuronal dysfunction. Based on these observations, it is hypothesized that S1P metabolism plays a key role in podocyte physiology, although the details of these mechanisms remain unclear.

Over the past years, extensive work has focused on S1P as a signaling molecule and it is now appreciated as a central bioactive lipid that regulates many fundamental physiological processes, including cell proliferation and survival, migration, vasculogenesis, and immune-cell trafficking [8,9,10]. S1P mainly acts as a ligand for high-affinity cell-surface receptors, denoted as S1P receptors (S1PR), of which five subtypes, S1P_1-5_, have been cloned and characterized [10,11,12]. Besides the extracellular action of S1P through S1PRs, intracellular S1P (iS1P) may also have signaling potential, although its direct targets are still elusive and controversial. 

S1P is generated by sphingosine kinases (Sphk) [13,14,15] and is irreversibly degraded by the ER-localized SPL [16,17] to produce phosphoethanolamine and hexadecenal, with the latter feeding back into the de novo biosynthetic pathway. 

The knockout of *Sgpl1* in mice was reported to cause kidney defects similar to those in *Pdgfrb*-deficient mice [18]. In these mice, the number and migration of glomerular mesangial cells were strikingly reduced [18], suggesting that *Sgpl1* is a platelet-derived growth factor (PDGF) target gene and regulates mesangial cell migration. Furthermore, *Sgpl1*-deficient mice had reduced viability and died by the age of 8 weeks [18]. Recently, the generation of an inducible *Sgpl1* knockout strain allowed a more detailed analysis of the kidney phenotype [19]. By electron microscopy analysis of the kidneys, these authors showed the typical foot process effacements of podocytes, increased matrix deposition in the mesangium, and tubular atrophy in the induced *Sgpl1* deficient mice [19] reminiscent of the phenotype seen in SPLIS patients. Exactly how this phenotype is regulated by accumulating iS1P on the molecular and cellular level is still unclear.

In the present study, we have used an immortalized human podocyte cell line to stably downregulate the SPL and to explore the molecular mechanisms by which SPL regulates podocyte function. We hypothesized that the loss of SPL would affect the proteins of the slit diaphragm complex, which are key for podocyte function in vivo. We found that SPL-kd podocytes show much lower mRNA and protein expression levels of nephrin and its regulating transcription factor, the Wilms tumor suppressor gene 1 (WT1), which may explain the podocyte dysfunction leading to proteinuria in vivo. In a step toward unraveling the mechanism, we have identified the specific protein kinase C (PKC)δ isoenzyme to be involved in the pathomechanism, because PKCδ phosphorylation, a measure of enzyme activation, was increased in the SPL-kd, and the selective knockdown of PKCδ in podocytes enhanced nephrin and WT1 expression. Furthermore, the proinflammatory cytokine, interleukin 6 (IL-6), also reduced nephrin expression and enhanced PKCδ phosphorylation. These data suggest that the phenotype of SPL mutations in vivo, which leads to steroid-resistant nephrotic syndrome, is due to the depressive effect of accumulating iS1P on WT1 and its target gene, nephrin, and that PKCδ is a key protein kinase, contributing to the pathology, and that selective inhibitors of PKCδ should be considered as a target for the pharmacological treatment of a steroid-resistant nephrotic syndrome induced by SPL mutations.

## 2. Results

### 2.1. Generation and Characterization of SPL Knockdown in Human Podocytes

**Figure 1:**
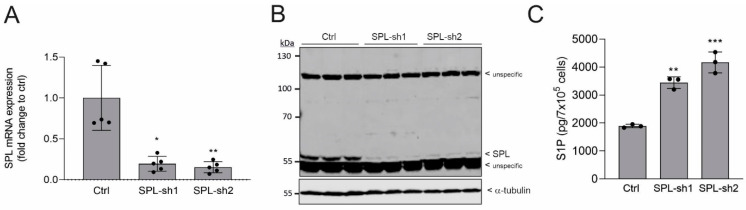
Characterization of stable SPL knockdown in human podocytes. Human podocytes that were stably transduced with an empty vector (Ctrl) or two different SPL shRNA constructs (SPL-sh1: TRCN0000078314; SPL-sh2: TRCN0000286832: further denoted as SPL-kd) were differentiated to mature podocytes for 5 days at 37 °C, then incubated for 4 h in serum-free medium. (**A**) RNA extracts were taken for quantitative PCR analysis of human SPL and 18S RNA for normalization. Data show the mRNA expression of SPL (Sgpl1) as a fold change, with means ± SD (*n* = 5). (**B**) Protein extracts were taken for SDS-PAGE, transferred to nitrocellulose, and subjected to Western blot analysis, using an antibody against SPL (dilution 1:1000, upper panel, SPL at 59 kDa) and α-tubulin for normalization (1:3000, lower panel). (**C**) Ctrl and SPL-kd cells were taken for lipid extraction and the mass spectrometric quantification of S1P. Data show cellular S1P concentrations and are expressed as pg/7 × 10^5^ cells, with means ± SD (*n* = 3). * *p* < 0.05, ** *p* < 0.01, *** *p* < 0.001 are considered statistically significant when compared to the Ctrl samples.

To elucidate the molecular mechanism by which SPL loss-of-function mutations or depletion triggers podocyte dysfunction and contributes to a nephrotic syndrome in vivo, we generated a stable SPL knockdown of an immortalized human podocyte cell line. Two shRNA constructs of SPL were tested for knockdown efficiency (Figure 1A). Both constructs revealed an 80–90% reduction of SPL mRNA (Figure 1A) and an almost complete depletion of the SPL protein (Figure 1B). Furthermore, both cell lines showed significantly increased cellular S1P levels (Figure 1C). For all subsequent studies, we concentrated on one cell line, i.e., SPL-sh2 (further denoted as SPL-kd).

### 2.2. SPL Knockdown Downregulates WT1 and Nephrin Expression in Podocytes

**Figure 2:**
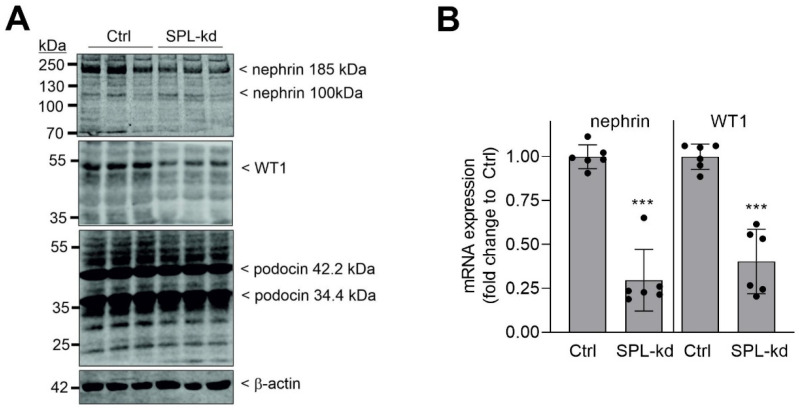
The effect of SPL-kd on nephrin and WT1 mRNA and protein expression in human podocytes. Human podocytes that were transduced with an empty vector (Ctrl) or a vector containing an SPL shRNA (SPL-kd), then differentiated to mature podocytes for 5 days at 37 °C, were incubated for 4 h in serum-free medium before the protein (**A**) and RNA (**B**) were extracted. Proteins were separated by SDS-PAGE, transferred to a nitrocellulose membrane, and subjected to Western blot analysis (**A**) of nephrin (top panel), WT1 (second panel), podocin (third panel) or β-actin (bottom panel). RNA were takenfor quantitative PCR analysis using the primers of human nephrin, WT1, and 18S RNA for normalization (**B**). Data in A show the details of one experiment performed in triplicate and is representative of at least three independent experiments. The bands corresponding to nephrin, WT1, podocin, and β-actin were evaluated using ImageJ software and are depicted in Supplementary Figure S2. The data in B are means ± SD (*n* = 3). *** *p* < 0.001 is considered statistically significant when compared to the Ctrl samples.

The SPL-kd cell line was further characterized for differences in the expression of the podocytic protein, nephrin, which is known to contribute to the ultrafiltration barrier in the kidney and has turned out to be a key factor that is downregulated in many forms of glomerular disease [20,21]. Western blot analysis revealed that the nephrin protein was decreased in SPL-kd cells (Figure 2A, upper panel). Notably, several bands are detected for nephrin, which derives from the fact that nephrin is a highly glycosylated protein and also exists as splice variants and fragments. According to the amino acid sequence, nephrin has a calculated mass of 135 kDa but runs at 185–200 kDa, due to multiple glycosylation sites [22]. In addition, cleavage may occur, under certain conditions, at amino acids 112 and 317, thus yielding bands of 124 kDa and 101 kDa, respectively [23]. The treatment of podocytes with tunicamycin, which, in general, blocks protein glycosylation, reduces the 185 kDa band of nephrin, first to a 135 kDa band and, at higher concentrations of tunicamycin, to a 100 kDa band (Supplementary Figure S1), which fits, in terms of size, with the previously described cleaved form of nephrin [23].

Since nephrin is critically regulated by the transcription factor WT1 [24,25], we also detected this factor using Western blot analysis. As seen in Figure 2A, in the lower panel, WT1 protein expression was also reduced in SPL-kd cells. In contrast, podocin, another member of the podocyte slit diaphragm complex, existing as two splice variants of 42.2 kDa and 34.4 kDa, was not altered by SPL-kd (Figure 2A, third panel). Quantitative PCR analysis was performed to see whether the reduced protein expression was derived from the reduced gene transcription of nephrin and WT1. Indeed, in the SPL-kd cells, both nephrin and WT1 mRNA steady-state levels were suppressed (Figure 2B). 

### 2.3. SPL Knockdown Stimulates Cellular PKC Activity in Podocytes

**Figure 3:**
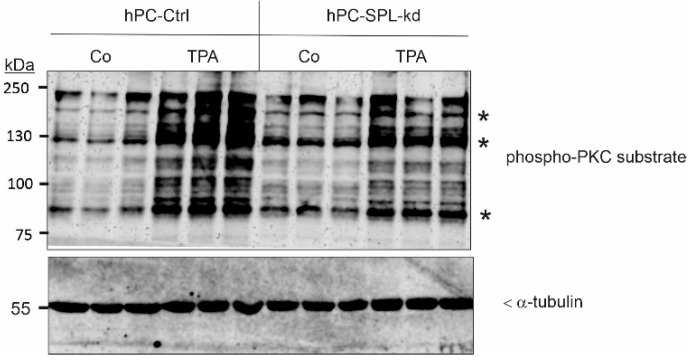
Effect of SPL-kd on cellular PKC activity in podocytes. Podocytes were treated for 15 min with either vehicle (DMSO, Co) or with 100 nM TPA, then taken for protein extraction and separation by SDS-PAGE, transferred to nitrocellulose, and subjected to Western blot analysis, using an anti-phospho-PKC substrate antibody (upper panel) and α-tubulin (lower panel). The data show one representative experiment in triplicate. Bands at 90 kDa, 130 kDa, and 190 kDa (indicated by *) were evaluated using the ImageJ software and are depicted in Supplementary Figure S3.

In a further attempt to unravel the mechanism by which SPL-kd could trigger the downregulation of WT1 and nephrin transcription, we investigated a key signaling enzyme, i.e., PKC, that could be affected by SPL-kd. In particular, our previous studies in cerebral endothelial cells revealed that SPL-kd resulted in enhanced cellular PKC activity [26]. To this end, the cells were stimulated for 15 min with a low concentration of the PKC activator, 12-O-tetradecanoyl-phorbol 13-acetate (TPA); total cellular PKC activity was detected in the Western blot analysis, using a phospho-PKC substrate antibody. TPA caused a strong increase of phosphorylation in various PKC substrates (bands at approximately 200, 190, 130, 120, 100, and 90 kDa) in both cell lines (Figure 3), which reflects the total activation of Ca^2+^-dependent, conventional PKCs and Ca^2+^-independent novel PKCs [27,28]. Notably, untreated SPL-kd cells showed the basally increased phosphorylation of a subset of bands at 190, 130, and 90 kDa, suggesting that SPL-kd, specifically, leads to the activation of one or several of the PKC isoenzymes. Some of the TPA-phosphorylated bands were rather reduced in SPL-kd. This may be explained by the fact that PKC activation leads to the rapid downregulation of the PKCs; an increased activation of PKC leads to faster downregulation, which then also manifests on the substrate level. 

**Figure 4:**
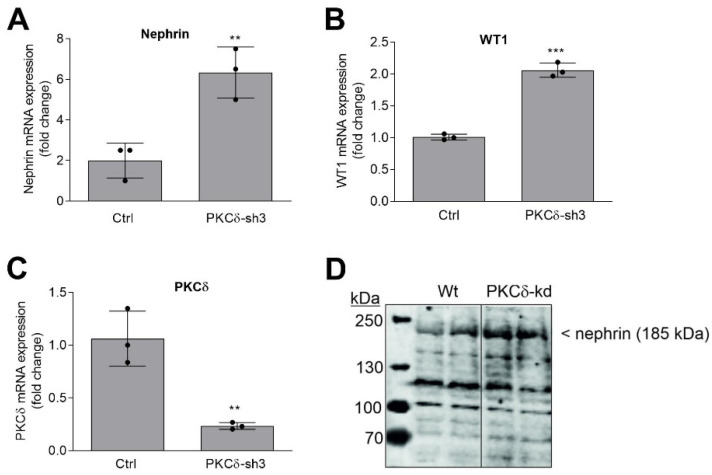
The effect of the stable knockdown of PKCδ on nephrin and WT1 mRNA and protein expression in podocytes. (**A**) Podocytes that were stably downregulated for PKCδ (PKCδ-sh3; PKCδ-kd) were taken for either RNA (**A**–**C**) or protein (**D**) extraction. RNA was used for the quantitative PCR analysis of nephrin (**A**), WT1 (**B**), and PKCδ (**C**) mRNA expression. 18S RNA was used for normalization. Results show the mRNA expression levels as fold change and are means ± SD (*n* = 3); ** *p* < 0.01 and *** *p* < 0.001 were considered statistically significant when compared to the Ctrl samples. Protein extracts were separated by SDS-PAGE, transferred to nitrocellulose, and subjected to Western blot analysis using an antibody against nephrin. The data show a representative blot in duplicates.

From the known PKC isoenzymes, PKCα and PKCδ, in particular, have previously been appointed a role in regulating albuminuria in mice with diabetic nephropathy [29,30]. In addition, our previous study had led us to hypothesize that PKCδ specifically contributed to the dysregulation of podocyte function by Sphk2-derived S1P [31]. In that study, we showed that only PKCδ-kd, but not PKCα-kd, enhanced the WT1 protein levels. Therefore, we have now concentrated on PKCδ. Three different shRNA constructs were tested; two of these shRNA constructs, sh3 and sh4, resulted in the strong downregulation of the PKCδ protein (Supplementary Figure S4). The stable cell line of PKCδ-sh3, further referred to as PKCδ-kd, was taken for the analysis of nephrin and WT1 mRNA expression. As shown in Figure 4A–C, nephrin and WT1 mRNA expression was upregulated when PKCδ was downregulated. In addition, the nephrin protein was also increased by PKCδ-kd (Figure 4D), which is congruent with the previous finding that the WT1 protein was increased by PKCδ-kd [31]. These data further strengthen the hypothesis that the loss or the inhibition of PKCδ exerts a protective effect on podocyte function.

### 2.4. Interleukin 6 Accelerates Nephrin Downregulation in SPL Knockdown Podocytes

**Figure 5:**
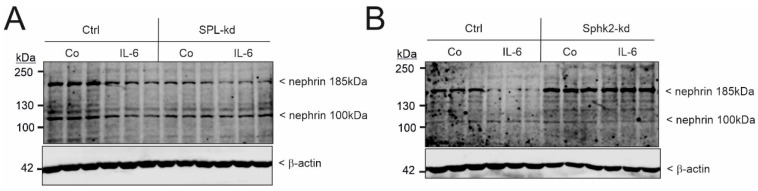
The effect of IL-6 on nephrin expression in SPL-kd and Sphk2-kd podocytes. (**A**) Podocytes that were either transduced with a control vector (Ctrl, **A**,**B**), SPL-kd (**A**), or Sphk2-kd (**B**) were treated for 24 h with either the vehicle (Co) or IL-6 (20 ng/mL). Protein extracts were taken for protein separation by SDS-PAGE, transferred to nitrocellulose, and subjected to Western blot analysis using antibodies against nephrin (upper panels) and β-actin (lower panels). Data show representative blots in triplicates. The corresponding bands were evaluated using ImageJ software and are depicted in Supplementary Figure S5.

Since it was previously reported that systemic SPL knockout mice show increased plasma levels of the proinflammatory cytokine, IL-6 [32], this also fosters an alternative hypothesis that the nephrotic syndrome seen in *Sgpl1^−/−^* (SPL knockout) mice and in *Sgpl1* mutant patients is mediated by a plasma factor and not necessarily by a podocyte-specific mechanism. Notably, podocytes are one of the few cell types that express the IL-6 receptor [33]. Therefore, we further tested whether exogenous IL-6 has an effect on podocyte nephrin expression. Indeed, we found that 24 h of IL-6 exposure caused a strong reduction in the nephrin protein (Figure 5A), which is consistent with the idea that IL-6 can disrupt the podocyte filtration barrier, leading to albuminuria, similar to that seen in SPL-kd. In contrast, podocytes with a stable Sphk2-kd showed an enhanced nephrin protein expression (Figure 5B), as also previously shown [31]. In these Sphk2-kd cells, nephrin suppression by IL-6 was abolished (Figure 5B). 

**Figure 6:**
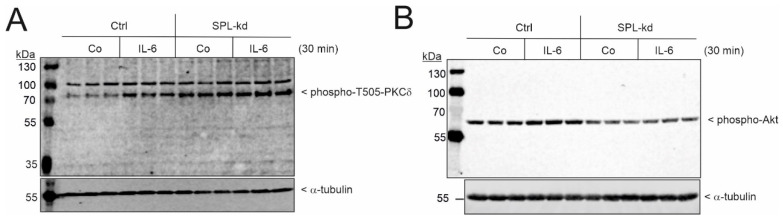
The effect of IL-6 on the phosphorylation and activation of PKCδ and Akt in Ctrl and SPL-kd podocytes. (**A**) Control transduced podocytes (Ctrl) or SPL-kd podocytes were treated for 30 min with either the vehicle (Co) or IL-6 (20 ng/mL). Protein extracts were taken for protein separation by SDS-PAGE, transferred to nitrocellulose, and subjected to Western blot analysis using antibodies against phospho-T^505^-PKCδ (**A**, upper panel), phospho-S^473^-Akt (**B**, upper panel), and α-tubulin (**A**,**B**, lower panels). The data show representative blots in triplicates. The corresponding bands were evaluated using ImageJ software and are depicted in Supplementary Figure S6.

Finally, we tested whether the observed IL-6-mediated suppression of nephrin involves PKCδ activation. To this end, cells were stimulated for 30 min with IL-6, then phospho-Thr^505^-PKCδ was detected by Western blot analysis. The Thr^505^ of PKCδ is an autophosphorylation site and is considered to coincide with PKCδ activity [34]. As seen in Figure 6A, IL-6 increases PKCδ autophosphorylation at this site. Moreover, in SPL-kd cells, the phosphorylation of PKCδ was constitutively higher, which is consistent with increased PKCδ activity. In contrast to the higher activation state of PKCδ in SPL-kd, the activity of Akt, as measured by the phosphorylation of Akt at Ser^473^, was reduced in SPL-kd (Figure 6B), suggesting a decreased survival capacity of the cells.

## 3. Discussion

Since the discovery that mutations in the *Sgpl1* gene, coding for S1P lyase (SPL), develop a severe form of steroid-resistant nephrotic syndrome [5,6,7], also classified as nephrotic syndrome type 14 (NPHS14), much attention has been directed to the role of iS1P in podocyte function. The renal pathology of NPHS14 closely resembles the pathology of *Sgpl1^−/−^* mice and includes not only albuminuria, podocyte effacement, and glomerulosclerosis but also tubular atrophy and tubulointerstitial fibrosis [19]. 

Our data here demonstrate for the first time that podocytes are, indeed, severely affected by the loss of SPL and respond with strongly downregulated nephrin expression. Nephrin is one of the key factors building the slit diaphragm and, thus, crucially regulates the glomerular ultrafiltration barrier. Many studies have, meanwhile, shown that the downregulation of nephrin in vivo leads to albuminuria, while the stabilization of nephrin is more renoprotective. Notably, Lovric et al. reported that in SPL-null *Drosophila* nephrocytes, the Neph1 homolog, Kirr, was unaffected compared to wild-type nephrocytes [5]. However, the authors did not further investigate other factors of the slit diaphragm. When using a human podocyte cell line or rat mesangial cells depleted of SPL by siRNA, only a decrease in mesangial cell migration was observed, but no effect was seen on mesangial cell apoptosis or proliferation, nor on podocyte migration or proliferation [5]. On the molecular level, SPL knockdown in mesangial cells showed reduced activities of the small G proteins, CDC42 and Rac1, which may explain the reduced migration seen in vitro, but cannot explain the glomerular phenotype seen in vivo [5].

Our data further show that SPL-kd in podocytes is associated with reduced WT1 expression. WT1 is centrally involved in nephrin transcription, and mutations in the *WT1* gene are also coupled to nephrotic syndrome. From this viewpoint, not only heterozygous WT1^+/−^ mice but also patients with one mutated *WT1* allele, have a high risk of developing renal failure. Two podocyte target genes for WT1 exist, which are nephrin and podocalyxin [24,35]. In mutated WT1 subjects, both proteins are downregulated; this correlates with podocyte foot process effacement and albuminuria. 

Not much is known about the regulation of WT1 transcriptional activation, although the WT1 promoter has previously been cloned [36,37]. Two transcription factors known to regulate WT1 are the hypoxia-inducible factors (HIF) 1 and 2 [37,38], which seems to be of special importance in the regulation of erythropoietin synthesis in erythropoietin producing cells [38]. PKCα and PKCδ have been shown to regulate HIF-1α on different levels [39]. Furthermore, it was shown that the aryl hydrocarbon receptor (AhR), which is a ligand-activated basic helix-loop-helix-PAS homology domain transcription factor, can alter WT1 splicing in the developing kidney, thereby disrupting renal differentiation and nephrogenesis [40]. AhR activation in mouse podocytes by indoxyl sulfate downregulates the various podocyte markers, including nephrin, podocin, podocalyxin, synaptopodin, and WT1 [41]. Notably, AhR has also been shown to be phosphorylated and activated by PKC [42]. Whether AhR is involved in the SPL-kd-mediated effect on WT1 and nephrin is presently unknown. 

Interestingly, PKC can also phosphorylate WT1 directly, which leads to the suppression of its DNA binding capacity [43]. Furthermore, Arellano-Rodriguez et al. [44] showed that in stained kidney sections from LPS-treated mice, WT1 was phosphorylated at two sites, i.e., Ser^393^ and Ser^363^, in podocytes and, upon phosphorylation, WT1 was translocated to the cytoplasm and lost its transcriptional activity. Both sites could represent PKA and/or PKC phosphorylation sites. These results would be in line with our findings that PKC activation results in reduced WT1 expression and, thus, also transcriptional activity. Besides these effects of PKC on transcription factors, PKC can also directly phosphorylate nephrin and trigger its internalization [45]. This process was shown to be mediated by PKCα. Since our data showed that PKCα-kd reduces nephrin and WT1 protein expressions, similar to SPL-kd, we rather exclude the theory that this isoenzyme is involved in the herein-observed nephrin downregulation. We suggest that this most probably occurs via a transcription mechanism that is mediated by PKCδ. That PKCδ contributes to diabetic nephropathy and albuminuria was also shown in a streptozotocin-induced diabetic nephropathy model, wherein PKCδ knockout mice had less albuminuria [30]. All these findings highlight the hypothesis that PKC is a master regulator of podocyte function, acting on different levels to guarantee the tight control of glomerular ultrafiltration.

PKCδ has also been attributed a key role in other inflammatory diseases, including acute and chronic kidney disease. In this regard, the inhibition of PKCδ reduced kidney injury in models such as cold storage ischemia-reperfusion-induced kidney injury [46], albumin overload-induced tubular injury [47], and left nephrectomy CKD [48]. On the other hand, patients with an autosomal recessive PKCδ deficiency suffered from early-onset autoimmunity, including systemic lupus erythematosus [49], and are prone to viral, bacterial, and fungal infections because of impaired ROS formation in phagocytic cells.

Based on our results, we herein propose the following mechanism: SPL-kd directly results in increased iS1P, which will activate PKCs, either directly or indirectly; among those is also the PKCδ isoenzyme, which then suppresses the WT1 activity that leads to reduced nephrin transcription. In this context, it has already been shown that SPL knockout (*Sgpl1^−/−^*) mice show increased diacylglycerol (DAG) levels in the liver [50] and in cultures of SPL-depleted fibroblasts and HeLa cells [51]. As DAG is a direct activator of PKC [52], this would explain the activating effect of SPL depletion on PKC. The mechanism of increased DAG in SPL-depleted cells and tissue is still incompletely understood. Based on the finding that the liver of *Sgpl1^−/−^* mice also showed increased sphingomyelin levels, it was proposed that the loss of SPL activates the conversion of ceramide to sphingomyelin by the action of sphingomyelin synthase. This enzyme transfers a phospho-choline group from phosphatidylcholine to ceramide, yielding not only sphingomyelin but also DAG as a side-product [50]. Alternatively, it could be envisioned that intracellular S1P directly activates PKCδ. Notably, S1P was shown to directly bind and activate PKCζ [53]. The binding site was characterized as a pocket on the surface of the kinase domain, reducing autoinhibitory contacts with the pseudosubstrate-C1 module to cause activation [53].

Another possible way in which SPL-kd mediates and triggers the downregulation of WT1 and nephrin is via another sphingolipid species, which, besides S1P, may also accumulate in SPL-kd [54]. For example, sphingosine is a well-known endogenous PKC inhibitor [55,56]. These early studies were performed with partially purified PKC to map total PKC activities and the Ca^2+^-dependent isoforms, which were the only known isoforms at that time. Meanwhile, several more PKC isoforms were identified, but the potential of sphingosine to inhibit all these novel isoforms was never systematically approached. However, Hamaguchi et al. [57] tested sphingosine in the context of PKCδ activity and found dose-dependent inhibition with an IC_50_ of 50 μM. This group also identified a novel sphingosine-dependent protein kinase (SDK), which was activated by sphingosine with an ED_50_ of approx. 10 μM. Interestingly, SDK turned out to be a 40 kDa cleavage product of PKCδ that was released by caspase 3 and contained the kinase domain of PKCδ [57]. The sphingosine-mediated inhibition of PKCδ has also been shown in mesothelioma cells, where it induced cell-cycle arrest and apoptosis (Okuwa et al., 2012).

Our previous study of Sphk2-kd podocytes also pointed to a key regulatory role of sphingosine in terms of nephrin expression and podocyte function. Sphingosine accumulated in Sphk2 cells, inhibited PKCδ, and thereby allowed WT1 transcriptional activation and the upregulation of nephrin mRNA and protein expression [31]. 

In addition to the effect of sphingosine/S1P alteration on podocyte function identified here, it was previously shown that the podocyte-specific depletion of acid ceramidase (*ASAH1*) in mice resulted in albuminuria and a nephrotic syndrome [58] that is very reminiscent of the SPL knockout phenotype. It is quite possible that an accumulation of ceramide, either lysosomal, as in ASAH1^−/−^, or non-lysosomal, as in Sgpl1^−/−^, is sufficient to trigger podocytopathy. 

Our data also demonstrate that the pro-inflammatory cytokine, IL-6, triggers nephrin downregulation, together with PKCδ activation. IL-6 is known to be increased systemically in many forms of chronic kidney disease [59]. IL-6 is also enhanced in the serum of *Sgpl1*-deficient mice [32], which develop glomerulosclerosis and tubular atrophy. Since podocytes are the only renal cell type that expresses the IL-6 receptor [33,60], one could speculate that systemic IL-6 directly participates in the podocyte dysregulation seen in *Sgpl1* mutations or deletion in vivo. Clearly, podocytes that are lacking SPL also change their function independently of IL-6; probably, both mechanisms work together to yield a severe form of renal dysfunction. However, how crucial the contribution of IL-6 may be to renal injury remains controversial. While one study in mice showed that the injection of recombinant IL-6 into mice was not sufficient to induce albuminuria or renal injury [60], a recent clinical study revealed the ameliorating effect of an IL-6 receptor antibody (tocilizumab) in a certain type of nephrotic syndrome, namely, Castleman disease [61]. 

On the molecular level, it was previously reported [62] that IL-6 contributes to podocyte injury by interrupting the podocyte’s focal adhesion dynamics and cytoskeletal organization; this involves STAT3 signaling. In addition, it was shown that IL-6 causes rapid PKCδ activation, which, in turn, directly phosphorylates STAT3 in HepG2 cells [63,64].

Altogether, our data have demonstrated that nephrin is a critical podocyte factor that is downregulated by the loss of SPL and may directly cause podocyte foot process effacement, as observed in mice and humans, leading to proteinuria as a hallmark of nephrotic syndrome. Furthermore, our in vitro data showed a key involvement of PKCδ in the downregulation of nephrin expression. Thus, PKCδ should be considered as a possible new target for the pharmacological treatment of a steroid-resistant nephrotic syndrome that is due to SPL mutations.

## 4. Materials and Methods

### 4.1. Chemicals

Dimethyl sulfoxide (DMSO), 12-O-tetradecanoyl-phorbol 13-acetate, tunicamycin, fatty acid-free bovine serum albumin (BSA), puromycin, the lentiviral particles of all shRNA constructs (MISSION^®^ shRNA), KAPA SYBR^®^ FAST, trypsin-EDTA 0.25%, horse serum*, RPMI-1640*, and Dulbecco’s modified Eagle medium (*DMEM)* containing 4.5 g/L glucose, were obtained from Sigma-Aldrich Fine Chemicals GmbH (Buchs, Switzerland). Fetal bovine serum (FBS) was purchased from PAN-Biotech GmbH (Catalog No. P40-37, Aidenbach, Germany). Fluorescently labeled Odyssee IRdye 800CW secondary antibodies were obtained from LI-COR Biosciences (Bad Homburg, Germany). Primers for quantitative PCR were obtained from Eurofins Genomics Germany GmbH (Ebersberg, Germany). The First Strand DNA Synthesis kit was obtained from ThermoFisher Scientific (Zug, Switzerland). RNAsolv^®^ was from VWR International AG (Dietikon, Switzerland). Human interleukin (IL) 6 was obtained from Peprotech (London, UK). All cell culture nutrients were from Life Technologies AG (Basel, Switzerland). The commercial antibodies were as follows: β-actin (clone AC-15) and α-tubulin (clone DM1A) were from Sigma-Aldrich Fine Chemicals GmbH (Buchs, Switzerland); WT-1 (C-19) was from Santa Cruz Biotechnology (Heidelberg, Germany); phospho-T^505^-PKCδ (cat. no. 9374), phospho-PKC substrate (cat. no. 2261), and phospho-Akt (Ser^473^) (cat. no. 4060) were from Cell Signaling Technology/BioConcept (Allschwil, Switzerland); PKCδ (cat. no. P36520) was obtained from the Transduction Laboratories/BD Biosciences (Allschwil, Switzerland). 

### 4.2. Peptide Synthesis and Polyclonal Antibody Generation

Peptide synthesis and anti-SPL antibody generation were performed by Eurogentec S.A. (Seraing, Belgium). Briefly, two synthetic peptides, ISA DTH KYG YAP KGS-C and C-TV TQG SQM NGS PKPH, were synthesized, based on an internal sequence and the C-terminal sequence of human SPL (Sgpl1; accession number: NM_003901). A cysteine was added to either the C-terminal or N-terminal end of the peptide to enable coupling to the keyhole-limpet hemocyanin. These coupled peptides were used in combination to immunize rabbits. The antibodies were purified by affinity chromatography, using an anti-peptide-coupled sepharose column for each peptide.

A human nephrin antibody (SA-1515) was generated, based on the two peptides (C-EYEESQWTGERDTQS and QPSGEPEDQLPTEPP-C) of human nephrin, and was characterized as previously described [65,66]. Human podocin antiserum (SA-1517) was generated, based on the two peptides (ERRARSSSRESRGRG-C and C-PVEPLNPKKKDSPML) of human NPHS2 (podocin) (accession number: NM_014625). 

### 4.3. Cell Culturing and Stable SPL and PKC Knockdown Generation

Immortalized human podocytes were isolated and cultivated at 33 °C, as previously described [67,68]. Prior to stimulation, cells at approximately 70% confluency were incubated for 5 days at 37 °C in a growth medium to allow the podocytes to differentiate. The cells were further incubated for 4 h in a serum-free medium and then stimulated in RPMI containing 10 mM Hepes, pH 7.4, and 0.1 mg/mL fatty acid-free bovine serum albumin (BSA), as indicated in the figure legends. The stable knockdown of SPL in hPC was achieved by the transduction of cells at 33 °C, with lentiviral constructs containing a short hairpin RNA (shRNA) against human SPL (Sigma MISSION^®^, TRCN0000078314 (SPL-sh1), TRCN0000286832 (SPL-sh2), TRCN0000195408 (PKCδ-sh2), TRCN0000010193 (PKCδ-sh3), and TRCN0000284800 (PKCδ-sh4), according to the manufacturer’s protocol. For all further experiments, the cell lines SPL-sh2 and PKCδ-sh3 were used, as denoted by SPL-kd and PKCδ-kd. The virus control cells were generated with TRC2 pLKO.5-puro empty vector control from the same company. For the selection of resistant colonies, 1 μg/mL of puromycin was added to the medium. The knockdown efficiencies were confirmed by quantitative PCR and Western blot analysis. The morphological change of podocytes up for differentiation for 7 days is shown in Supplementary Figure S7. Cells increased in size and showed a more irregular shape in terms of differentiation, but no difference was seen between the control cells and the SPL-kd cells. 

### 4.4. Western Blot Analysis

Stimulated cells were homogenized and processed exactly as previously described [31,69]. The nitrocellulose membranes were taken for Western blot analysis, using IR-coupled goat anti-rabbit and mouse 800 secondary antibodies. Multi-color Western blots were imaged by the Odyssey^®^ imaging system using 700 nm and 800 nm channels and then visualized using the ImageStudio software, version 5.2 (LICOR Biosciences, Bad Homburg, Germany). For the transfer of nephrin to nitrocellulose, 12 V for 1.5 h was applied in a semi-dry blotting machine (Bio-Rad Laboratories GmbH, Basel, Switzerland), with a transfer buffer containing 25 mM Tris base, 190 mM glycine, 20% (*v*/*v*) methanol, and 0.03% (*w*/*v*) SDS. 

### 4.5. RNA Extraction and Quantitative PCR Analysis

The stimulated cells were washed with ice-cold PBS and homogenized in RNA-Solv^®^ reagent. Total RNA extraction was performed according to the instructions from the manufacturer. The yield and purity of the isolates were assessed with a NanoDrop^®^ ND-1000 spectrophotometer (Witec AG, Littau, Switzerland), and first-strand cDNA was synthesized using 2 μg of total RNA as a template. SYBR^®^ Green-based quantitative PCR was performed in a BioRad CFX Connect™ Optics Module thermal cycler (Bio-Rad Laboratories Inc., Hercules, FL, USA) The Bio-Rad CFX Manager software was used to monitor the melting curve and to obtain the quantification data. The relative mRNA expression of the gene of interest was calculated using the ΔΔCt method, normalized to 18S RNA as a housekeeping gene. The primers used were as follows: hSGPL1 (SPL) forward: ATAGATCCTGTCCCTGAAGT, reverse: CACCTTTCACCCGGAAATCA; hWT1: forward: ATAACCACACAACGCCCATC; reverse: TCAGATGCCGACCGTACAAG. hNPHS1 (nephrin) forward: CAACTGGGAGAGACTGGGAGAA; reverse: AATCTGACAACAAGACGGAGCA, hPRKCD (PKCδ) forward: GCCAAGGTGTTGATGTCTGTT, reverse: CCCACTGTTGTCTTGCATGTC.

### 4.6. Quantification of S1P by LC-MS/MS

Cell monolayers in dishes of 60 mm in diameter were trypsinized, pelleted, and resuspended in methanol containing internal standards; they were then subjected to lipid extraction and LC-MS/MS analysis, exactly as previously described [31,70].

### 4.7. Statistical Analysis

Statistical analysis was performed via a one-way ANOVA, without matching or an unpaired *t*-test, where applicable. For multiple comparisons, the level of significance was calculated with a Bonferroni correction. GraphPad Prism 8.4.3 Software (La Jolla, CA, USA) was used for statistical analysis and graph representations.

## Data Availability

The authors declare that all data supporting the findings of this study are available either within this paper or in the Supplementary Data File, or they can be obtained from the corresponding author upon request.

## Supplementary Materials

The supporting information can be downloaded at: https://www.mdpi.com/article/10.3390/ijms24043267/s1.

## Abbreviations

AhR	Arylhydrocarbon receptor
DAG	Diacylglycerol
IL-6	Interleukin 6
Kd	knockdown
HIF	hypoxia-inducible factor
LC-MS	liquid chromatography-mass spectrometry
PDGF	platelet-derived growth factor
PKC	protein kinase C
S1P	sphingosine 1-phosphate
SDS-PAGE	sodium dodecyl sulfate-polyacrylamide gel electrophoresis
shRNA	small hairpin RNA
SphK	sphingosine kinase
SPL, Sgpl1	S1P lyase
SPLIS	S1P lyase insufficiency syndrome
TPA	12-O-tetradecanoyl-phorbol 13-acetate
WT1	Wilms tumor suppressor gene 1
