# Supplementary material for: Loss of S1P Lyase Expression in Human Podocytes Causes a Reduction in Nephrin Expression That Involves PKCδ Activation
Source: Int J Mol Sci. 2023 Feb 7;24(4):3267. doi: 10.3390/ijms24043267 (PMC9965238; doi:10.3390/ijms24043267)
Supplement: Supplementary file 1 [file ijms-24-03267-s001.zip › ijms-2160389-supplementary.pdf]

Supplementary data:

Manuscript title: Loss of S1P lyase expression in human podocytes causes a reduction of nephrin expression that involves PKCδ activation.

Authors: Imeri, Stepanovska Tanturovska, Manaila, Pavenstädt, Pfeilschifter, and Huwiler

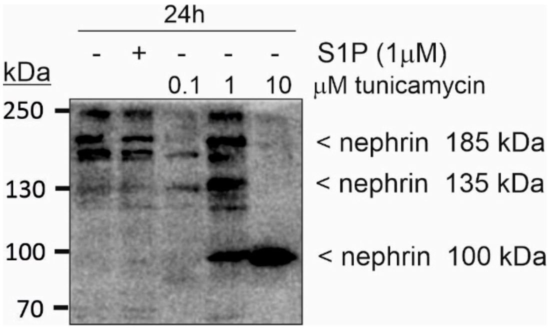

Figure S1: Effect of tunicamycin on nephrin protein expression in wildtype human podocytes. Human podocytes were treated for 24 h with either vehicle (-) or S1P (+), or the indicated concentrations of tunicamycin to block protein glycosylation. Protein lysates, containing 80 µg of protein were separated by SDS-PAGE, transferred to nitrocellulose and subjected to Western blot analysis using antibodies against nephrin at a dilution of 1:1000.

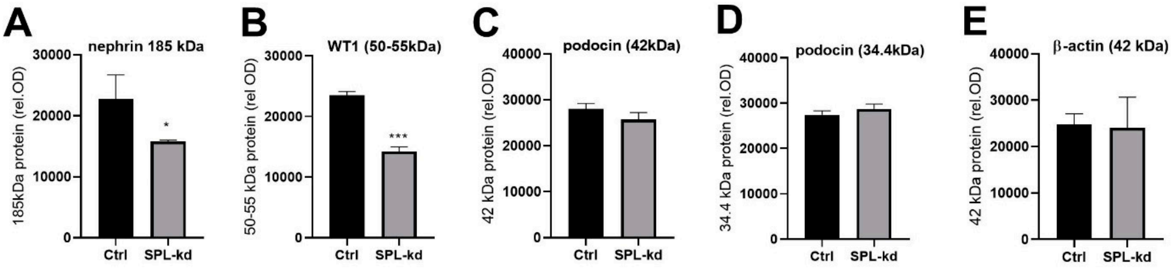

Figure S2

Western blots presented in Fig. 2A were evaluated by ImageJ software. Results show the relative change of nephrin (A), WT1 (B), the two podocin isoforms 42 kDa (C) and 34.4 kDa (D), and β-actin (E), between Ctrl and SPL-kd cells. Data are presented as means ± S.D. (n=3). *p<0.05, ***p<0.001 compared to the Ctrl samples.

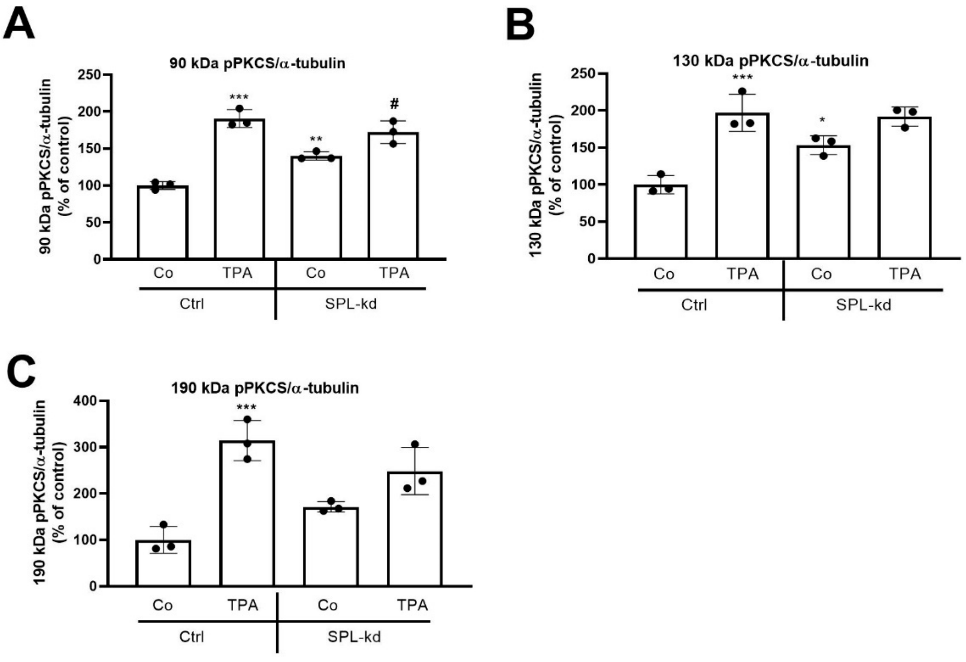

Figure S3

The blot presented in Fig. 3 was evaluated by ImageJ software. Results show the relative change of the different PKC substrates at 90 kDa (A), 130 kDa (B), and 190 kDa (C) when normalized to α-tubulin. Data are presented as means ± S.D. (n=3). *p<0.05, **p<0.01, ***p<0.001 compared to the Ctrl-Co samples; #p<0.05 compared to the Ctrl-TPA samples.

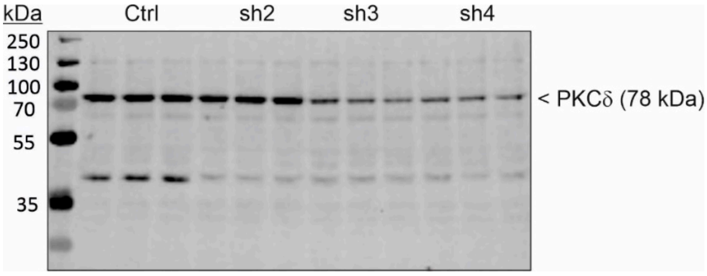

Figure S4

Human podocytes were transduced with lentiviral shRNA constructs of PKCδ (sh2, sh3, sh4) or an empty vector (Ctrl). Stable cell lines in triplicates were taken for protein extraction, protein separation by SDS-PAGE, transfer to nitrocellulose and Western blot analysis using an antibody against PKCδ (Transduction Laboratories, 1:1000)

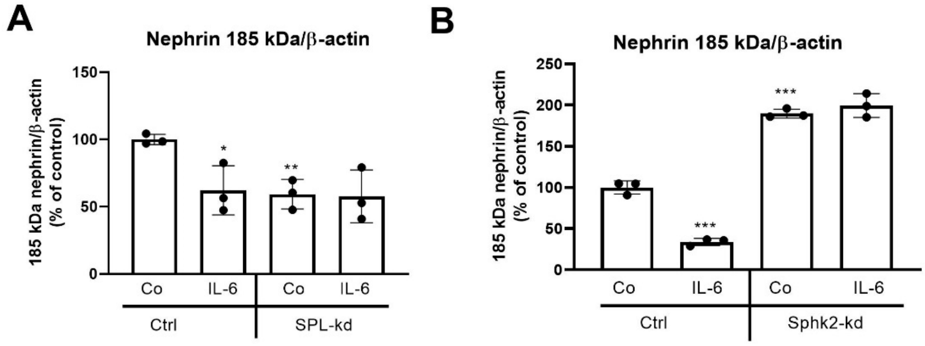

Figure S5

The blots presented in Fig. 5A and 5B were evaluated by ImageJ software. Results show the relative change of the 185 kDa nephrin band when normalized to β-actin. Data are presented as means ± S.D. (n=3). *p<0.05, **p<0.01, ***p<0.001 compared to the Ctrl-Co samples.

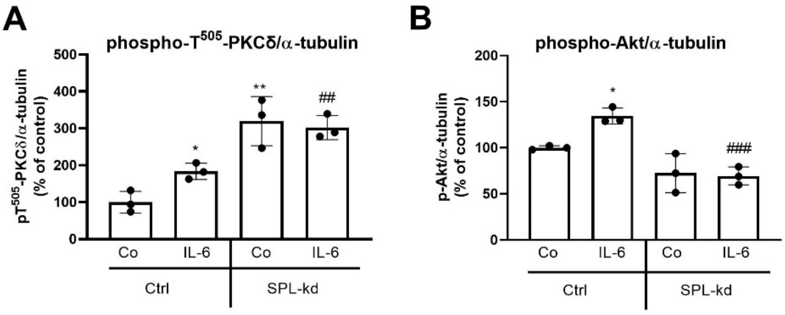

Figure S6

The blots presented in Fig. 6A and 6B were evaluated by ImageJ software. Results show the relative change of the 78 kDa phospho-T^{505}-PKCδ (A) or phospho-Akt (B) normalized to α-tubulin. Data are presented as means ± S.D. (n=3). *$p<0.05$, ***$p<0.001$ compared to the Ctrl samples; ##$p<0.01$, ###$p<0.001$ when compared to the Ctrl-IL-6 samples.

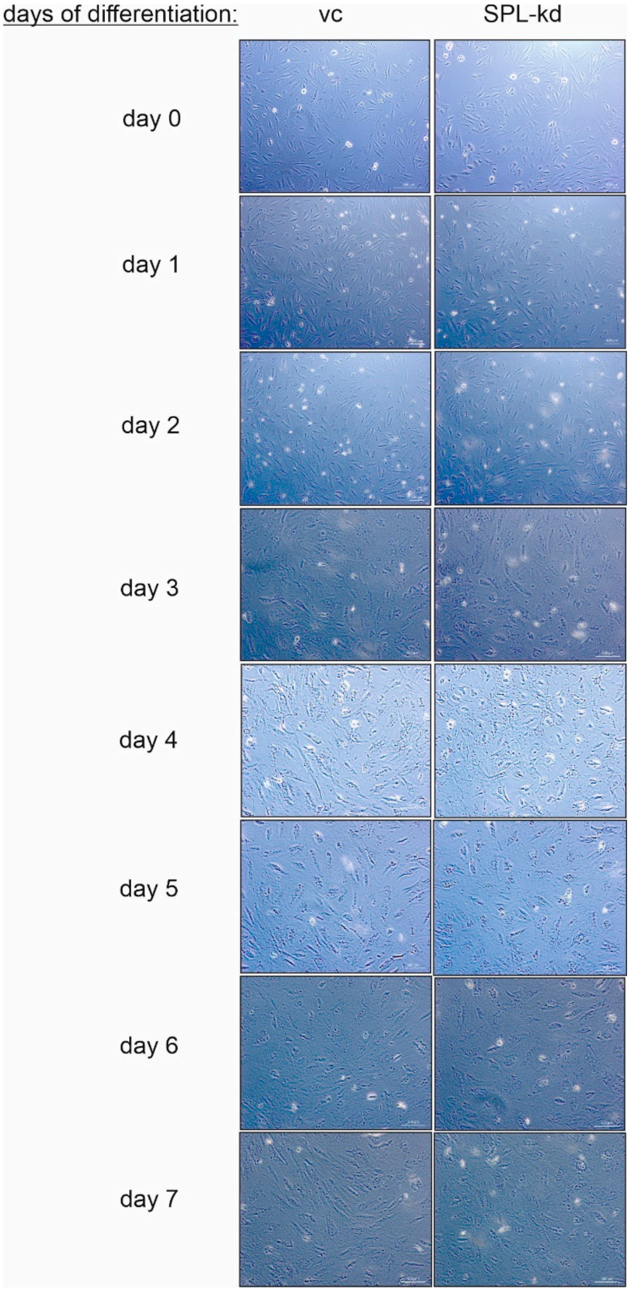

Figure S7

Control transduced human podocytes (vc) or stable SPL-kd podocytes at day 0 until day 7 of differentiation at 37°C, were taken for a light microscopic picture using a Zeiss AxioObserver Z1 (Feldbach, Germany) microscope with phase contrast setting. Scale bars, 100µm.
